# The RNA-induced transcriptional silencing complex targets chromatin exclusively via interacting with nascent transcripts

**DOI:** 10.1101/gad.292599.116

**Published:** 2016-12-01

**Authors:** Yukiko Shimada, Fabio Mohn, Marc Bühler

**Affiliations:** 1 Friedrich Miescher Institute for Biomedical Research, 4058 Basel, Switzerland;; 2 University of Basel, 4003 Basel, Switzerland

**Keywords:** heterochromatin, RNAi, noncoding RNA, epigenetics, nascent transcript

## Abstract

Small RNAs regulate chromatin modification and transcriptional gene silencing across the eukaryotic kingdom. Although these processes have been well studied, fundamental mechanistic aspects remain obscure. Specifically, it is unclear exactly how small RNA-loaded Argonaute protein complexes target chromatin to mediate silencing. Here, using fission yeast, we demonstrate that transcription of the target locus is essential for RNA-directed formation of heterochromatin. However, high transcriptional activity is inhibitory; thus, a transcriptional window exists that is optimal for silencing. We further found that pre-mRNA splicing is compatible with RNA-directed heterochromatin formation. However, the kinetics of pre-mRNA processing is critical. Introns close to the 5′ end of a transcript that are rapidly spliced result in a bistable response whereby the target either remains euchromatic or becomes fully silenced. Together, our results discount siRNA–DNA base pairing in RNA-mediated heterochromatin formation, and the mechanistic insights further reveal guiding paradigms for the design of small RNA-directed chromatin silencing studies in multicellular organisms.

## Abstract (precis)

In this study, Shimada et al. use fission yeast to investigate how small RNA-loaded Argonaute protein complexes target chromatin to mediate silencing and show that transcription of the target locus is essential for RNA-directed formation of heterochromatin. Their results provide new mechanistic insights into small RNA-directed chromatin silencing.

RNAi broadly refers to silencing pathways that depend on conserved Argonaute family proteins to repress gene expression (Höck and Meister 2008). A unifying feature of all RNAi pathways is that Argonaute-bound small RNAs guide protein effector complexes to complementary targets to mediate gene silencing. Although very diverse in composition, these are commonly termed RNA-induced silencing complexes (RISCs) (Pratt and MacRae 2009). Besides mediating sequence-specific degradation or translational repression of target mRNAs (PTGS), small RNAs are also implicated in chromatin modification and transcriptional gene silencing (TGS) in ciliates, fungi, plants, and the germline of animals (Malone and Hannon 2009; Mochizuki 2010; Castel and Martienssen 2013). Pioneering studies in plants and the fission yeast *Schizosaccharomyces pombe* have provided a conceptual framework for studying the molecular mechanisms of these conserved small RNA-directed chromatin silencing pathways (Moazed 2009; Matzke and Mosher 2014). However, due to technical limitations, some of their most fundamental mechanistic aspects have remained obscure. For most systems studied so far, it is unclear whether the siRNA-loaded RISC targets the DNA or the nascent transcript of a complementary locus. To address this, we made use of the well-understood fission yeast RNAi pathway.

The single *S. pombe* Argonaute protein (Ago1) is at the core of the *S. pombe* RISC, known as the RNA-induced transcriptional silencing (RITS) complex (Verdel et al. 2004). RITS is loaded predominantly with small RNAs complementary to centromeric repeats and is essential for heterochromatin formation at centromeres (Verdel et al. 2004). Upon target recognition, RITS recruits the RNA-dependent RNA polymerase (RdRP) complex (RDRC), which synthesizes dsRNA using the targeted transcript as a template. This results in additional substrate for siRNA processing by the ribonuclease Dcr1, establishing a positive feedback loop that confers stability to constitutive heterochromatin (Motamedi et al. 2004; Colmenares et al. 2007). Methylated histone H3 Lys9 (H3K9) is a conserved hallmark of heterochromatin (Allis and Jenuwein 2016). This chromatin modification is installed by the Clr4 complex (CLRC) (Hong et al. 2005; Horn et al. 2005; Jia et al. 2005), which is recruited to the target locus by RITS (Bayne et al. 2010; He et al. 2013). Clr4 is essential for setting up a stable RNAi-induced heterochromatin domain and is notably the sole *S. pombe* histone H3K9 methyltransferase.

Central to the understanding of RNAi-directed heterochromatin formation is how RITS is targeted to chromatin. The current view is that siRNA-programmed RITS initially recognizes the nascent transcript of its target locus. However, this has not been demonstrated directly. It is supported by the observation that both RITS and the RDRC can be cross-linked to centromeric RNA as well as to DNA (Motamedi et al. 2004). This suggests that RITS is targeted to chromatin through base pairing between siRNA and pre-mRNA followed by recruitment of RDRC and histone-modifying enzymes (Bühler et al. 2006; Bühler and Moazed 2007). However, it does not rule out base pairing of siRNAs also with the target locus DNA. Moreover, it has remained elusive whether pre-mRNAs are targeted by siRNA-programmed RITS and what impact, if any, mRNA splicing has on the kinetics of heterochromatin formation.

In wild-type *S. pombe* cells, siRNAs do not trigger the formation of stable heterochromatin at euchromatic genes in *trans* (Sigova 2004; Bühler et al. 2006). This has hampered a systematic mechanistic dissection of siRNA-mediated chromatin silencing. We recently discovered that siRNA-directed heterochromatin formation in *S. pombe* is inhibited by the RNA polymerase-associated factor 1 complex (Paf1C). Paf1C mutant strains are highly susceptible to de novo assembly of heterochromatin and stable gene silencing by synthetic, *trans*-acting primary siRNAs complementary to the coding sequence of protein-coding genes (Kowalik et al. 2015). This has provided us with a unique tool to address these remaining fundamental mechanistic questions. In this study, we demonstrate for the first time that RITS cannot target DNA in *S. pombe*. We also found that transcription above a minimal threshold is obligatory for small RNA-directed heterochromatin formation but that high transcriptional activity at an siRNA target locus counteracts heterochromatin assembly. We further show that pre-mRNA splicing is compatible with RNAi-directed heterochromatin formation and that intronic sequences can serve as binding sites for siRNAs that are acting in *trans*. Together, our results provide the first direct evidence for the nascent transcript model of small RNA-directed epigenetic gene repression and reveal guiding paradigms for the design of small RNA-directed chromatin silencing experiments in other organisms.

## Results

### siRNAs complementary to intronic sequences trigger formation of heterochromatin

Current models of RNAi-directed heterochromatin assembly propose that RISC/RITS is targeted to chromatin via base-pairing interactions with nascent or chromatin-bound RNAs (Motamedi et al. 2004; Nakama et al. 2012; Holoch and Moazed 2015). However, it has not been demonstrated that truly nascent transcripts (i.e., unspliced pre-mRNAs) can be targeted by siRNA-programmed RITS for the formation of heterochromatin in fission yeast.

**Figure 1.**
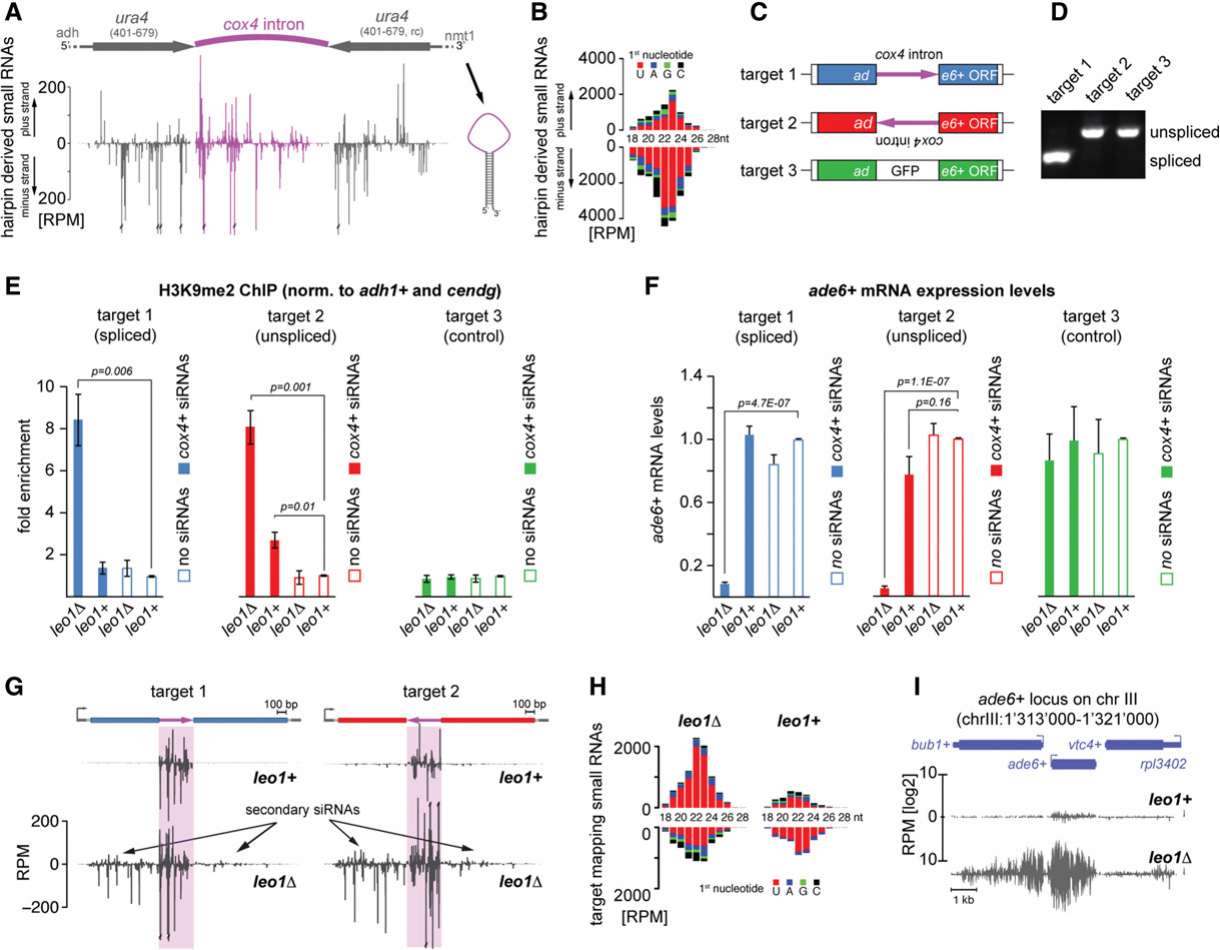
Transcription start site (TSS)-distal pre-mRNA splicing does not affect the stability of heterochromatin. (*A*) Schematic of the *adh1*^+^ promoter-driven *ura4* hairpin construct. The double-stranded stem consists of complementary *ura4*^+^ ORF sequences (401–679; gray), while the loop encodes the *cox4*^+^ intron (pink) (Iida et al. 2008). Normalized 5′ ends of derived siRNAs are depicted *below* as reads per million (RPM). (*B*) Length histogram of generated siRNAs from *A* colored by their 5′ starting nucleotides. (*C*) Schematic representation of siRNA target constructs. The *cox4*^+^ intron sequence was inserted at position +700 nt of the *ade6*^+^ ORF in forward or reverse orientation (targets 1 and 2, respectively). A GFP fragment of the same size but without canonical splice sites was inserted at the same position (target 3). (*D*) RT–PCR to assess intron removal was performed with primers flanking the exon–exon junction (mb2202 and mb167). (*E*) Chromatin immunoprecipitation (ChIP) experiments with an antibody recognizing H3K9me2. Fold enrichments were normalized to *adh1*^+^ and *cendg* and are shown relative to the respective *leo1*^+^/no siRNA samples. Error bars indicate SD. *n* = 3 independent biological replicates. Two-tailed Student's *t*-test. (*F*) *ade6*^+^ mRNA levels were determined by quantitative RT–PCR. Values were normalized to *act1*^+^ mRNA and are shown relative to the respective *leo1*^+^/no siRNA samples. Error bars indicate SD. *n* = 3 independent biological replicates. Two-tailed Student's *t*-test. (*G*) Normalized siRNAs mapping to the spliced (target 1; blue) and unspliced (target 2; red) targets in *leo1*^+^ (*top*) and *leo1*Δ (*bottom*) cells. Primary siRNAs produced from the *cox4*^+^ intron in the hairpin (shown in *A*) are shaded in pink. Secondary siRNAs are generated solely at the target locus. (*H*) Length histogram of target mapping siRNAs, similar to *B*. (*I*) Browser screen shot depicting siRNAs mapping to the *ade6*^+^ target locus.

To test this directly, we used *S. pombe* strains in which the *leo1*^+^ gene was deleted. Leo1 is a protein subunit of Paf1C, which inhibits siRNA-directed assembly of heterochromatin at euchromatic genes (Kowalik et al. 2015). In addition, we used a synthetic RNA hairpin construct that is expressed from the *nmt1*^+^ locus, driven by an *adh1*^+^ promoter (Fig. 1A; Iida et al. 2008). This construct contains a 355-nucleotide (nt) intron from the *cox4*^+^ gene, separating inverted repeats that are complementary to *ura4*^+^ and encode the stems of the hairpin (Fig. 1A). Besides siRNAs originating from the double-stranded *ura4*^+^ sequence in the hairpin, comparable amounts of siRNA reads are generated from the *cox4*^+^ intron in the loop of the hairpin construct (Fig. 1A; Yu et al. 2014; Kowalik et al. 2015). The hairpin-derived small RNAs are, on average, 23 nt in length and predominantly start with a uridine, confirming that they are bona fide siRNAs (Fig. 1B; Supplemental Fig. S1).

We showed previously that the intronless *ade6*^+^ gene is highly susceptible to siRNA-directed assembly of heterochromatin in Paf1C mutant but not wild-type cells (Kowalik et al. 2015). To assess the impact of splicing on the formation of heterochromatin at the endogenous *ade6*^+^ locus, we inserted the *cox4*^+^ intron sequence in the middle of the *ade6*^+^ ORF by homologous recombination. To discriminate between exonic and intronic targeting, we inserted the *cox4*^+^ intron in either forward or reverse orientation (target 1 and target 2, respectively). Insertion of a GFP sequence of similar length but devoid of any canonical splice sites (target 3) served as the control for siRNA specificity (Fig. 1C). RT–PCR analysis of total RNA revealed effective *ade6*^+^ splicing if the *cox4*^+^ intron is transcribed in its forward orientation (Fig. 1D). As expected, the *cox4*^+^ intron in reverse orientation or the GFP fragment did not enable *ade6*^+^ pre-mRNA splicing.

Consistent with our previous results, we observed methylation of H3K9 at the nonspliced locus only in the presence of complementary hairpin-derived *cox4* siRNAs (Fig. 1E, target 2). In contrast, in the absence of sequence complementarity to the siRNA in target 3, H3K9 methylation was not induced irrespective of the presence of *cox4* siRNAs (Fig. 1E). Importantly, we also observed *cox4* siRNA-directed H3K9 methylation when the siRNA targeted region is spliced in target 1 (Fig. 1E). The level of H3K9 methylation was the same at both the spliced and nonspliced target loci, and the *ade6*^+^ mRNA levels of both targets were strongly reduced upon siRNA-directed H3K9 methylation (Fig. 1F). Furthermore, in *leo1*Δ but not *leo1*^+^ cells, secondary siRNAs were generated at the targeted *ade6*^+^ locus (Fig. 1G; Supplemental Fig. S2), similar to our previous findings when providing *ade6* siRNAs (Kowalik et al. 2015). These secondary small RNAs have the characteristic siRNA signature (Fig. 1H), and their spreading is highly directional, as expected, due to the activity of RDRC. Notably, besides generating abundant secondary siRNAs toward the 5′ end of the *ade6*^+^ locus, spreading extends even several kilobases beyond, including the *bub1*^+^ gene (Fig. 1I). Thus, intron-targeting primary siRNAs are sufficient to effectively initiate silencing of *ade6^+^,* including recruitment of RDRC. These results unequivocally show that pre-mRNA splicing is compatible with RNAi-directed heterochromatin formation and that intronic sequences can serve as binding sites for siRNAs that act in *trans*.

### Promoter-proximal introns are inferior to distal introns

**Figure 2.**
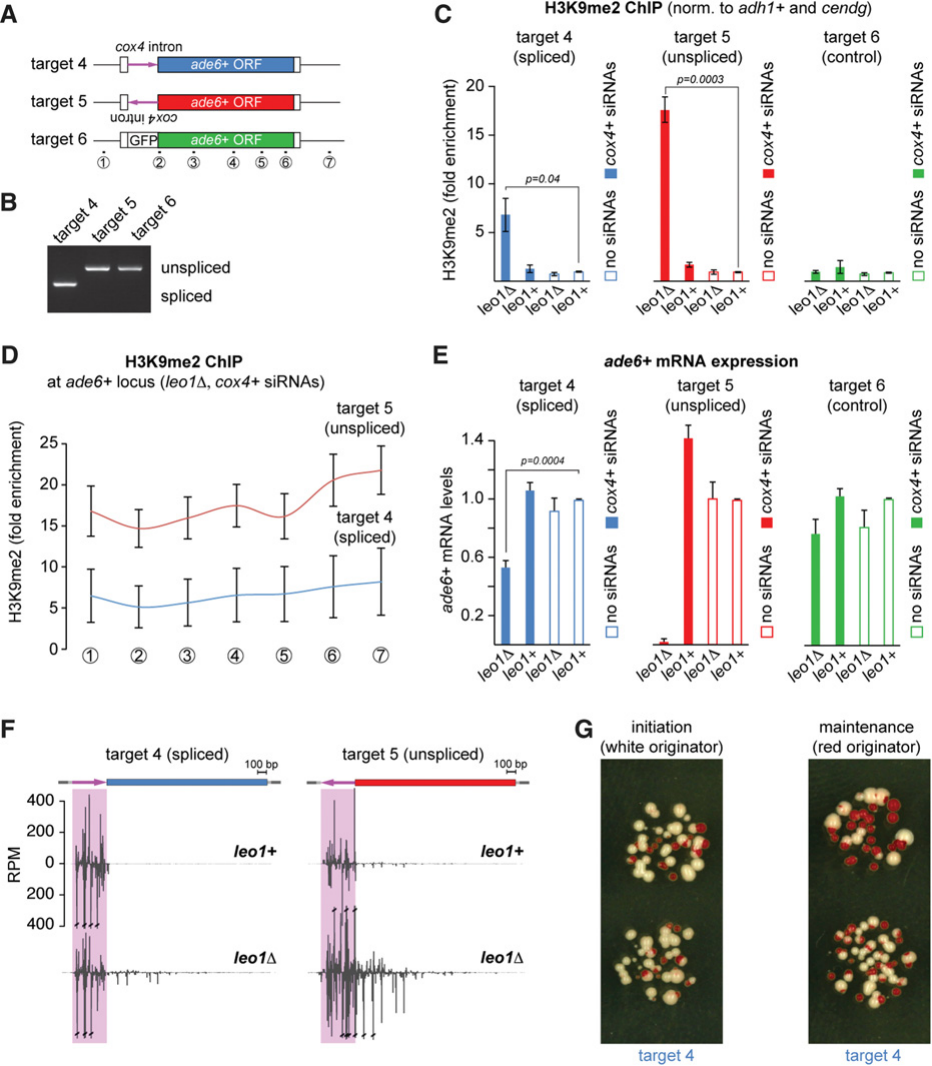
TSS-proximal pre-mRNA splicing reduces the stability of heterochromatin. (*A*) Schematic representation of siRNA target constructs. The *cox4*^+^ intron (in forward and reverse orientation in targets 4 and 5, respectively) and GFP control sequence were inserted 46 nt after the TSS of the endogenous *ade6*^+^ gene. Numbers indicate the positions of primer pairs used in *D*. (*B*) RT–PCR to assess intron removal was performed with primers flanking the exon–exon junction (mb10007 and mb10008). (*C*) ChIP experiments with an antibody recognizing H3K9me2. Fold enrichments were normalized to *adh1*^+^ and *cendg* and are shown relative to the respective *leo1*^+^/no siRNA samples. Error bars indicate SD. *n* = 3 independent biological replicates. Two-tailed Student's *t*-test. (*D*) H3K9me2 enrichment across the *ade6*^+^ locus in *cox4* siRNA-expressing *leo1*Δ cells. The primer pairs used are indicated in *A*. Error bars indicate SD. (*E*) *ade6*^+^ mRNA levels were determined by quantitative RT–PCR. Values were normalized to *act1*^+^ mRNA and are shown relative to the respective *leo1*^+^/no siRNA samples. Error bars indicate SD. *n* = 3 independent biological replicates. Two-tailed Student's *t*-test. (*F*) Normalized siRNA mapping to the spliced (target 4; blue) and unspliced (target 5; red) targets, similar to Figure 1G. (*G*) *leo1*Δ cells expressing the spliced siRNA target gene (target 4) and *cox4* primary siRNAs were seeded on yeast extract (YE) plates. White and red originator colonies were spotted on yeast extract plates to assess the initiation and maintenance of silencing, respectively.

The above results suggest that RNA splicing does not prevent RNAi-directed heterochromatin assembly per se. To test whether this also applies to splicing events at the very beginning of a transcript that presumably occur immediately after the nascent transcript emerges, we generated strains in which the *cox4*^+^ intron was inserted 46 nt after the annotated transcription start site of the *ade6*^+^ gene (Fig. 2A). As before, we inserted the *cox4*^+^ intronic sequence in both orientations (targets 4 and 5) as well as a GFP fragment of similar length as a specificity control (target 6) and confirmed splicing by RT–PCR (Fig. 2B).

In contrast to target constructs 1 and 2 (Fig. 1), we observed significant differences in the amount of H3K9 methylation and *ade6*^+^ mRNA silencing between the spliced and nonspliced targets 4 and 5. Although H3K9 methylation was induced at both loci specifically by the *cox4* siRNAs, H3K9me2 levels were inherently lower if the *cox4*^+^ target sequence underwent splicing (Fig. 2C,D). Consistent with this observation, *ade6*^+^ silencing was *cox4* siRNA-specific and very robust without splicing of the *cox4*^+^ intronic sequence (targets 5 and 6) (Fig. 2E). However, *ade6*^+^ mRNA levels were reduced only twofold for the spliced target gene (target 4) (Fig. 2E), indicating that excision of the siRNA target sequence by an early splicing event obstructs heterochromatin formation. Despite the decreased silencing potential, RDRC-dependent secondary siRNAs were generated in a *leo1*Δ-dependent manner also at this target locus. Concomitantly, low levels of H3K9 methylation spanned the entire locus (Fig. 2D,F; Supplemental Figs. S1, S2). These observations allow two alternative interpretations: (1) Rapid splicing of the target sequence decreases the overall silencing potential and therefore only mildly reduces target expression in all cells. (2) Alternatively, due to early splicing, the initiation of silencing becomes stochastic, and only a fraction of cells trigger the full response, while others do not initiate silencing at all. Notably, H3K9me2, siRNAs, and RNA expression were all measured at the population level, integrating over millions of cells. Thus, if 50% of cells expressing the spliced target remained in a euchromatic state while the other half became heterochromatic, maximal twofold repression can be expected from such a population average. Therefore, we decided to address this question at single-cell resolution by growing cells on limiting adenine indicator plates, which leads to red colonies when *ade6*^+^ is fully repressed. Indeed, when single cells were seeded, ∼50% formed red colonies, indicative of complete *ade6*^+^ repression (Fig. 2G, left). This indicates that the kinetics of pre-mRNA processing is a critical parameter for heterochromatin formation. If the siRNA target is available only briefly, such as our spliced target 4, full assembly of the silencing machinery may occur in only a subset of cells, resulting in a bistable on/off state. Of note, once established, silencing is propagated through mitosis, albeit maintenance fidelity is lower than for targets without early splice events (Fig. 2G, right; Kowalik et al. 2015).

In sum, siRNA-binding sites close to the 5′ end of a transcript perform as well as more distal sites unless removed by RNA splicing. Although heterochromatin is established with low frequency when promoter-proximal introns are targeted, it is less stably maintained through mitosis (Fig. 2G). These results strongly argue against RITS invading the double-stranded underlying DNA or base-pairing with the ssDNA exposed in an R loop upon transcription (Nakama et al. 2012), as, in both cases, splicing is not expected to affect heterochromatin formation.

### Highly transcribed genes resist siRNA-directed H3K9 methylation

**Figure 3.**
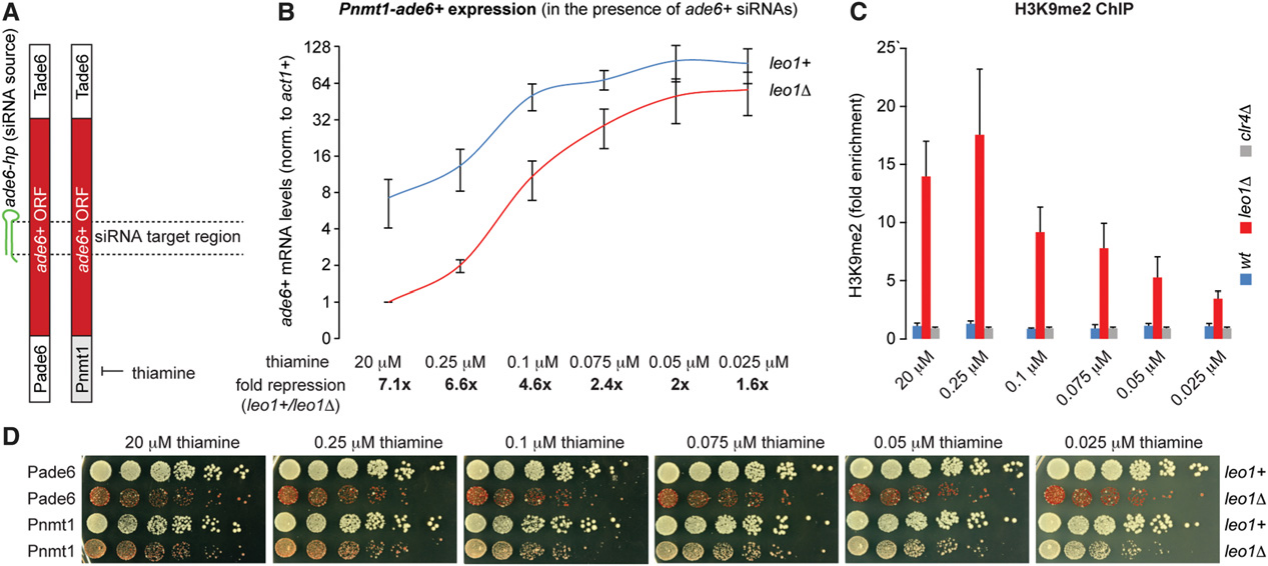
High transcriptional activity counteracts RNAi-directed heterochromatin assembly. (*A*) Scheme depicting the endogenous *ade6*^+^ gene (*left*) or the thiamine-repressible *nmt1* promoter (*right*). The siRNA target region is indicated by dashed lines. (*B*) *ade6*^+^ mRNA levels were determined by quantitative RT–PCR. Values were normalized to *act1*^+^ mRNA and are shown relative to *leo1*Δ cells grown in the presence of 20 µM thiamine. Error bars indicate SD. *n* = 3 independent biological replicates. (*C*) ChIP experiments with an antibody recognizing H3K9me2. Fold enrichments over *clr4*Δ cells are indicated. Error bars indicate SD. *n* = 3 independent biological replicates. (*B*,*C*) ChIP and quantitative RT–PCR experiments were performed with cells from the same culture. (*D*) Fivefold serial dilutions were spotted on adenine-limited PMG agar plates supplemented with thiamine at the concentrations indicated. Precultures were grown in YES medium, washed, and diluted in H_2_O before spotting.

The above results strongly support the hypothesis that RITS is targeted to chromatin via base-pairing interactions with nascent transcripts. However, they still do not completely rule out siRNA–DNA base pairing (Moazed et al. 2006). According to the latter model, *trans*-acting siRNAs are expected to assemble heterochromatin also in the absence of transcription at the target locus. Therefore, we investigated to what extent the deposition of H3K9 methylation is dictated by promoter strength of the target locus. For consistency, we chose the endogenous *ade6*^+^ gene as the siRNA target locus and first replaced its promoter with the strong but tunable *nmt1* promoter (Fig. 3A). When cells are grown on thiamine-containing medium, the nmt1 promoter is gradually repressed with increasing concentrations yet is not switched off completely (Fig. 3B, *leo1*^+^ cells; Forsburg 1993). As a source of *trans*-acting primary *ade6*^+^ siRNAs, we used a previously described stably integrated synthetic *ade6*^+^ RNA hairpin construct that is driven by an *adh1*^+^ promoter (Kowalik et al. 2015).

When grown under repressed conditions with high concentrations of thiamine, *ade6* siRNAs efficiently triggered H3K9 methylation specifically in *leo1*Δ cells (Fig. 3C). As a consequence, *ade6*^+^ mRNA levels were reduced up to sevenfold in *leo1*Δ compared with *leo1*^+^ cells (Fig. 3B,C). However, we observed a gradual reduction in silencing and H3K9 methylation efficiency with increasing expression of the target locus (Fig. 3B,C). Consistent with this, *leo1*Δ cells expressing *ade6*^+^ driven by the *nmt1* promoter formed red colonies on limiting adenine indicator plates supplemented with 20 µM thiamine, indicating full *ade6*^+^ repression (Fig. 3D). We also observed attenuated repression with decreasing thiamine concentrations for the *nmt1*-driven *ade6*^+^ but not for *ade6*^+^ driven by its own promoter (Fig. 3D). In contrast, *leo1*^+^ cells formed white colonies, demonstrating that repression was siRNA-specific and not caused by high concentrations of thiamine. *nmt1-ade6*^+^ expression in wild-type cells is still sufficient even when reduced at high concentrations of thiamine (Fig. 3B).

These results reveal that high transcriptional activity at an siRNA target locus can counteract heterochromatin assembly. This may be due to increased nucleosome turnover, leading to the loss of K9 methylated H3 or to RITS/Clr4 displacement from chromatin. Both scenarios would affect the positive feedback loop and therefore prevent stable formation of heterochromatin. Intermediate to low promoter activity should thus be considered as an important criterion in the design of small RNA-directed chromatin silencing experiments.

### Transcription activity above a minimal threshold is necessary for heterochromatin formation

**Figure 4.**
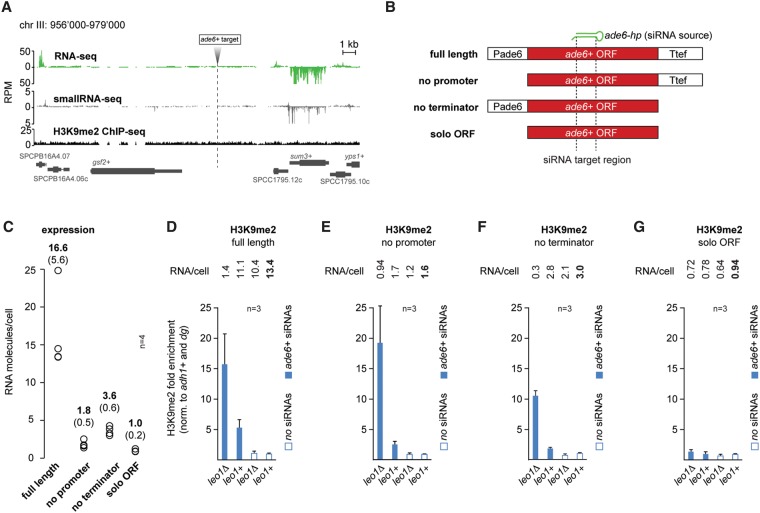
Transcription is a prerequisite for heterochromatin formation. (*A*) Genome browser screenshot showing the insertion site for the *ade6*^+^ transgenes depicted in *B*. Normalized RNA-seq (green), small RNA-seq (gray), and H3K9me2 ChIP-seq (ChIP combined with high-throughput sequencing) (black) tracks from wild-type cells, including annotated genes (*bottom*), are indicated. (*B*) Schematic representation of the *ade6*^+^ transgenes inserted on chromosome III at the positions indicated in *A*. The green hairpin denotes primary *ade6* siRNAs that are expressed from chromosome I (Kowalik et al. 2015). (*C*) The absolute number of *ade6* RNA molecules per cell was determined by droplet digital PCR (ddPCR) in *leo1*^+^ cells not expressing *ade6* siRNAs. *n* = 4 biological replicates. Average numbers of RNA molecules per cell are indicated in bold for each transgene, and standard deviation is shown in the brackets. (*D*–*G*) ChIP experiments were performed with an antibody recognizing H3K9me2 and the strains indicated. Enrichments were normalized to *adh1*^+^ and *cendg* and are shown relative to *leo1*^+^ cells that do not express *ade6* siRNAs. *n* = 3 independent biological replicates. Error bars indicate SD. Absolute numbers of *ade6* RNA molecules per cell were determined in one of the three ChIP replicates.

Because the *nmt1* expression system does not switch off completely, we aimed to abolish transcription of the endogenous *ade6*^+^ gene entirely by deleting its promoter. However, we still observed substantial residual transcriptional activity (data not shown), preventing us from distinguishing between the DNA and nascent RNA targeting models at the endogenous *ade6*^+^ locus. We reasoned that the residual *ade6*^+^ activity emanated from neighboring gene activities. Therefore, we examined the *S. pombe* genome for a region with no apparent sign of transcription to serve as a neutral landing site where transgenes would be less influenced by transcription occurring in the vicinity. Inspecting RNA sequencing (RNA-seq) and small RNA-seq data generated from wild-type *S. pombe* cells, we found a 17-kb region on chromosome III with no discernible RNA production. Furthermore, H3K9 methylation was not significantly enriched in this area (Fig. 4A). At that locus, we inserted different *ade6*^+^ transgenes: the full-length *ade6*^+^ gene with its own promoter and a terminator sequence, *ade6*^+^ without the promoter but with the terminator, *ade6*^+^ without the terminator but with the promoter, and the *ade6*^+^ ORF alone (Fig. 4B).

To determine the absolute number of *ade6*^+^ mRNA molecules per cell produced from these transgenes, we used the droplet digital PCR (ddPCR) technology. This revealed that the full-length *ade6*^+^ transgene driven by its own promoter gives rise to ∼17 RNA molecules per cell. For the transgene lacking the promoter, we counted only 1.8 RNA molecules per cell, while the transgene without transcription termination signals produced 3.6 *ade6*^+^ RNA molecules per cell. For the solitary *ade6*^+^ ORF (solo ORF) inserted at the same position, we never counted more than one molecule per cell (Fig. 4C). Thus, as expected, in the absence of transcriptional regulatory elements, expression of *ade6*^+^ is very low or highly infrequent.

If RITS targeted DNA, *trans*-acting *ade6* siRNAs would be expected to trigger H3K9 methylation at all four transgenes. We detected H3K9 methylation at the full-length *ade6*^+^ gene, at *ade6*^+^ without the promoter, and at *ade6*^+^ without the terminator (Fig. 4D–F). However, we did not observe methylated H3K9 at the *ade6*^+^ solitary ORF transgene when less than one transcript per cell was present (Fig. 4G). Together with the sensitivity to splicing (Fig. 2), these results demonstrate that the RITS complex cannot bind to DNA. We therefore conclude that RITS is targeted to chromatin exclusively via base-pairing interactions with nascent transcripts. The absence of any H3K9 methylation despite residual transcription activity at the solitary *ade6*^+^ ORF indicates that synthesis of a minimal number of nascent transcripts must be achieved to recruit sufficient amounts of chromatin-modifying enzymes.

## Discussion

### siRNA–DNA interactions do not initiate de novo assembly of heterochromatin

Originating from initial discoveries in yeast and plants, the role of RNAi-related pathways in epigenetic genome regulation has triggered much excitement. It provides an attractive mechanistic explanation for the largely elusive question of how chromatin-modifying enzymes find their targets. Current models state that the sequence information contained in the loaded small RNAs guides enzymatic activities to complementary targets via base-pairing interactions with nascent RNAs (Grewal 2010; Allshire and Ekwall 2015; Martienssen and Moazed 2015). However, sequence-specific interactions involving siRNA–DNA base pairing have not been refuted. The following key observations from the data presented here rule out that targeting RITS to DNA is sufficient to initiate the formation of silent chromatin in *S. pombe*: (1) Transcription above a certain threshold is an absolute requirement for the formation of heterochromatin. Silencing does not initiate if less than one RNA molecule is present per cell (Fig. 4). (2) Splicing of siRNA-binding sites close to the 5′ end of a transcript interferes with heterochromatin assembly (Fig. 2). If siRNAs targeted RITS to DNA, heterochromatin stability between spliced and nonspliced targets would not be different. Therefore, we conclude that RITS is programmed by Dcr1-produced siRNAs to target specific chromosomal regions exclusively via siRNA nascent transcript base-pairing interactions.

### Conservation of the nascent transcript model

There is accumulating evidence that the nascent transcript model applies to small RNA-directed chromatin silencing pathways also in other organisms. The strongest support comes from studies in *Drosophila*, where piRNAs in follicle cells originate almost 100% from the antisense strand of transposable elements. These trigger silencing only if the target harbors complementary piRNA sites in sense orientation. Because silencing of reporters and endogenous loci is strictly dependent on the orientation of the piRNA targets, direct DNA targeting as well as targeting of ssDNA exposed in R loops can be excluded also in flies (Sarot et al. 2004; Sienski et al. 2012; Post et al. 2014).

However, Argonaute engagement with the underlying DNA in other systems likewise awaits direct disproof. For example, RNA-directed DNA methylation (RdDM), a major small RNA-mediated epigenetic pathway in plants, requires a specialized transcriptional machinery that comprises the two plant-specific RNA polymerases: polymerase IV (Pol IV) and Pol V (Matzke and Mosher 2014). Pol IV transcribes precursors that are processed by an RdRP (RDR2) and DCL3 (DICER-LIKE 3) to produce siRNAs that load onto AGO4 (Herr et al. 2005). Pol V produces transcripts that are widely accepted to act as scaffolds for association of AGO4–siRNA complexes and subsequent chromatin modification (Wierzbicki et al. 2008). However, although current models depict siRNA base-pairing with Pol V-generated scaffold RNAs, it is still elusive whether guide RNAs bind directly to DNA exposed by Pol V transcription or to nascent transcripts produced from it (Dalakouras and Wassenegger 2014; Matzke and Mosher 2014). Consistent with our results in *S. pombe*, 24-nt secondary siRNAs induce DNA methylation in *trans* at unlinked target sites only if this sequence is transcribed by RNA Pol II. However, methylation can nevertheless occur without detectable transcription at this target in the presence of 21- to 24-nt hairpin-derived siRNAs. This suggests that synthesis of a nascent transcript at some target loci is not essential for RdDM and that methylation of nontranscribed target sequences in *Arabidopsis* may require multiple size classes of siRNA (You et al. 2013). The nascent transcript model has also been put forward to explain small RNA-directed chromatin regulation in *Caenorhabditis elegans* (Grishok 2005; Guang et al. 2010; Burkhart et al. 2011; Buckley et al. 2012; Luteijn et al. 2012; Wedeles et al. 2013). Whether siRNA–DNA base pairing is negligible in these processes has not been addressed. However, conservation of nascent transcript targeting in fission yeast and *Drosophila* suggests that this is an evolutionarily conserved mode of inducing chromatin modifications.

### Epigenetic silencing is a digital process

Our efforts in triggering the formation of heterochromatin by *trans*-acting primary siRNAs in *S. pombe* highlight a key feature of epigenetic gene regulation. Rather than inducing gradually decreasing mRNA levels, siRNAs initiate gene silencing in an “all or nothing” fashion in Paf1C mutant cells. Once established, the off state is stably propagated (Kowalik et al. 2015) unless the siRNA complementary sequence is situated in an early intron (Fig. 2). Similar digital silencing responses occur upon artificial tethering of various chromatin-modifying enzymes to a transcriptional reporter in mammalian cells (Bintu et al. 2016). This type of silencing is physiologically highly relevant, as exemplified by the regulation of the *FLOWERING LOCUS C* (*FLC*) in *Arabidopsis*. *FLC* expression is gradually repressed by prolonged cold exposure when measured at the level of a tissue. However, this seemingly “analog” silencing response is the net result of digital silencing events that occur in individual cells of that particular tissue over time. Thereby, a population of cells as a whole can respond quantitatively to an environmental change by simply switching individual cells from an on state to an off state (Berry and Dean 2015).

We infer that the siRNA-induced off switch is triggered as soon as the local concentration of chromatin-modifying activities and their residence time on the target are sufficiently high with respect to the target transcript itself. Only if this ratio is above a certain threshold will the epigenetic changes take effect and be maintained. Thus, future efforts should focus on quantifying such thresholds under physiological conditions for small RNA target interactions as well as for other epigenetic modifiers. This will not only further our understanding of epigenetic gene silencing but hopefully also enable us to generate meaningful predictions of putative targets for epigenetic regulation based on steady-state measurements.

Finally, we note that current state-of-the-art biochemical techniques interrogating cell populations are not well-suited for quantitative analysis of epigenetic gene regulation. As exemplified in this study, epigenetic silencing in individual cells may be easily missed if gene expression is assessed at the population level. Hence, adjusting current and developing new methods at single-cell resolution will be important for future mechanistic studies.

## Materials and methods

### Strains and plasmids

Fission yeast strains were grown at 30°C in YES medium. All strains were constructed following a PCR-based protocol (Bahler et al. 1998) or by standard mating and sporulation. Strains generated in this study are in Supplemental Table 2.

### Silencing assay

To assess *ade6*^+^ expression, serial fivefold dilutions of the respective strains were plated on yeast extract plate or PMG plates supplemented with 5.65 mg/L adenine and 226 mg/L each leucine, urail, histidine, and lysine.

### RNA isolation and cDNA synthesis

RNA was isolated using Absolutely RNA miniprep kit (Agilent) as described in Emmerth et al. (2010). cDNA was synthesized using PrimeScript RT Master Mix (Takara).

### Quantitative real-time PCR

Real-time PCR on cDNA samples and chromatin immunoprecipitation (ChIP) DNA was performed as described in Emmerth et al. (2010) using a Bio-Rad CFX96 real-time system using SsoAdvanced SYBR Green supermix (Bio-Rad). Primer sequences are in Supplemental Table 1.

### Qualitative RT–PCR

PCR on cDNA was performed using a fast-cycling PCR kit (Qiagen). PCR products were analyzed by agarose gel electrophoresis. Primer sequences are in Supplemental Table 1.

### ddPCR

Exponentially growing cells were harvested, and the cell number was determined with a hemocytometer (Thoma). Typically, 1.2 × 10^7^ cells were used to isolate RNA using the MasterPure yeast RNA purification kit (Epicenter). Two-hundred nanograms of total RNA isolated from mouse embryonic stem cells was added to the yeast samples in lysis buffer to estimate the recovery rate of RNA. cDNA was synthesized using the PrimeScript RT Master Mix (Takara). cDNA corresponding to 20 ng of RNA was used as template for ddPCR. The PCR reaction was prepared with QX200 ddPCR EvaGreen supermix (Bio-Rad) and Droplet generation oil for EvaGreen (Bio-Rad). Droplets were generated with a QX200 droplet generator (Bio-Rad). After amplification to the end point, droplets were quantitated by QX200 droplet reader (Bio-Rad).

### ChIP

ChIP experiments were performed as described previously in Kowalik et al. (2015) with histone H3K9me2-specific mouse monoclonal antibody from Wako (clone no. MABI0307).

### Small RNA-seq

Small RNA libraries were prepared as described previously (Kowalik et al. 2015). In brief, total RNA was isolated, and small RNAs (18–28 nt) were size-selected by PAGE. Libraries were prepared using the Illumina TruSeq small RNA preparation protocol according to manufacturer's instructions. Libraries were sequenced on an Illumina HiSeq2500 instrument.

### Small RNA-seq data analysis

Cutadapt (Cutadapt –a “adapter” --discard-untrimmed -m 18) was used to remove the 3′ adaptor form the raw reads. Reads <18 nt and untrimmed reads were removed. Trimmed reads were aligned to the *S. pombe* genome (ASM294 version 2.24) using Bowtie (Langmead et al. 2009). No mismatches were allowed, and, for multimapping reads, one random best hit was kept (-M 1 -v 0 --best --strata). For mapping the reads to the hairpin, similar settings were used due to the inverted *ura4* sequence in the hairpin stem (see Fig. 1A). To map the reads to the targets, only uniquely mapping reads were allowed (-m 1 -v 0 --best --strata). Mapping stats for all samples are in Supplemental Figure S3.

### Accession numbers

Small RNA-seq data have been deposited at the NCBI Gene Expression Omnibus (GEO) database and are accessible through GEO series number GSE87672.

## Supplementary Material

Supplemental Material
