# Supplementary material for: The RNA-induced transcriptional silencing complex targets chromatin exclusively via interacting with nascent transcripts
Source: Genes Dev. 2016 Dec 1;30(23):2571–80. doi: 10.1101/gad.292599.116 (PMC5204350; doi:10.1101/gad.292599.116)
Supplement: Supplemental Material [file supp_30_23_2571__index.html]

The RNA-induced transcriptional silencing complex targets chromatin exclusively via interacting with nascent transcripts — The RNA-induced transcriptional silencing complex targets chromatin exclusively via interacting with nascent transcripts — Supplemental Material 

# The RNA-induced transcriptional silencing complex targets chromatin exclusively via interacting with nascent transcripts

## Supplemental Material

- Supplemental\_Information\_FINAL.doc
