# Supplementary material for: The RNA-induced transcriptional silencing complex targets chromatin exclusively via interacting with nascent transcripts
Source: Genes Dev. 2016 Dec 1;30(23):2571–80. doi: 10.1101/gad.292599.116 (PMC5204350; doi:10.1101/gad.292599.116)
Supplement: Supplemental Material [file supp_gad.292599.116_Supplemental_Information_FINAL.doc]

**SUPPLEMENTAL INFORMATION**

**The RNA-induced transcriptional silencing complex targets chromatin exclusively via interacting with nascent transcripts**

Yukiko Shimada, Fabio Mohn, and Marc Bühler

**Supplemental Data**


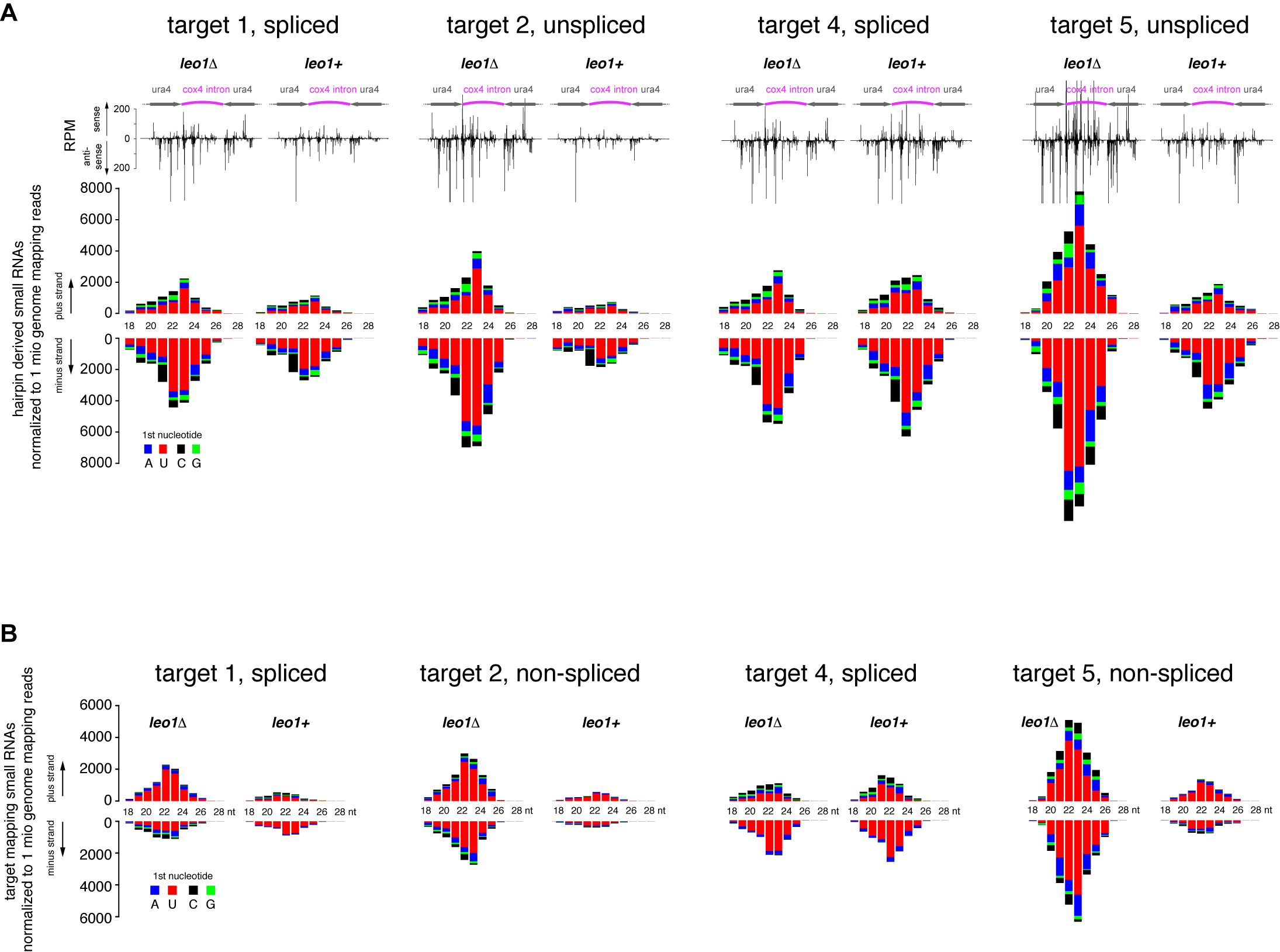


**Figure S1.** (**A**) Top: Normalized mapped 5’ ends of siRNAs originating from the ura4 hairpin constructs in *leo1+* and *leo1Δ* cells from all smallRNA sequencing data sets. Depicted as reads per million (RPM). Bottom: Length histograms of hairpin siRNAs colored by their 5’ starting nucleotide. (**B**) Similar to (A, bottom), but analysing siRNAs mapping to the respective target constructs.

**Figure S2.** Normalized browser screenshots for loci targeted by hairpin-derived siRNAs. The centromere region of chromosome III (left) serves as control to show that centromeric siRNA production is comparable in all experiments irrespective of genotype and hairpin construct. Note the secondary siRNA spreading specific to *leo1Δ* in all samples at the *ade6+* and *cox4*+ loci. Of note, the *ura4+* locus is deleted in the strains used, therefore the hairpin derived ura4 siRNAs have no taget in the genome.


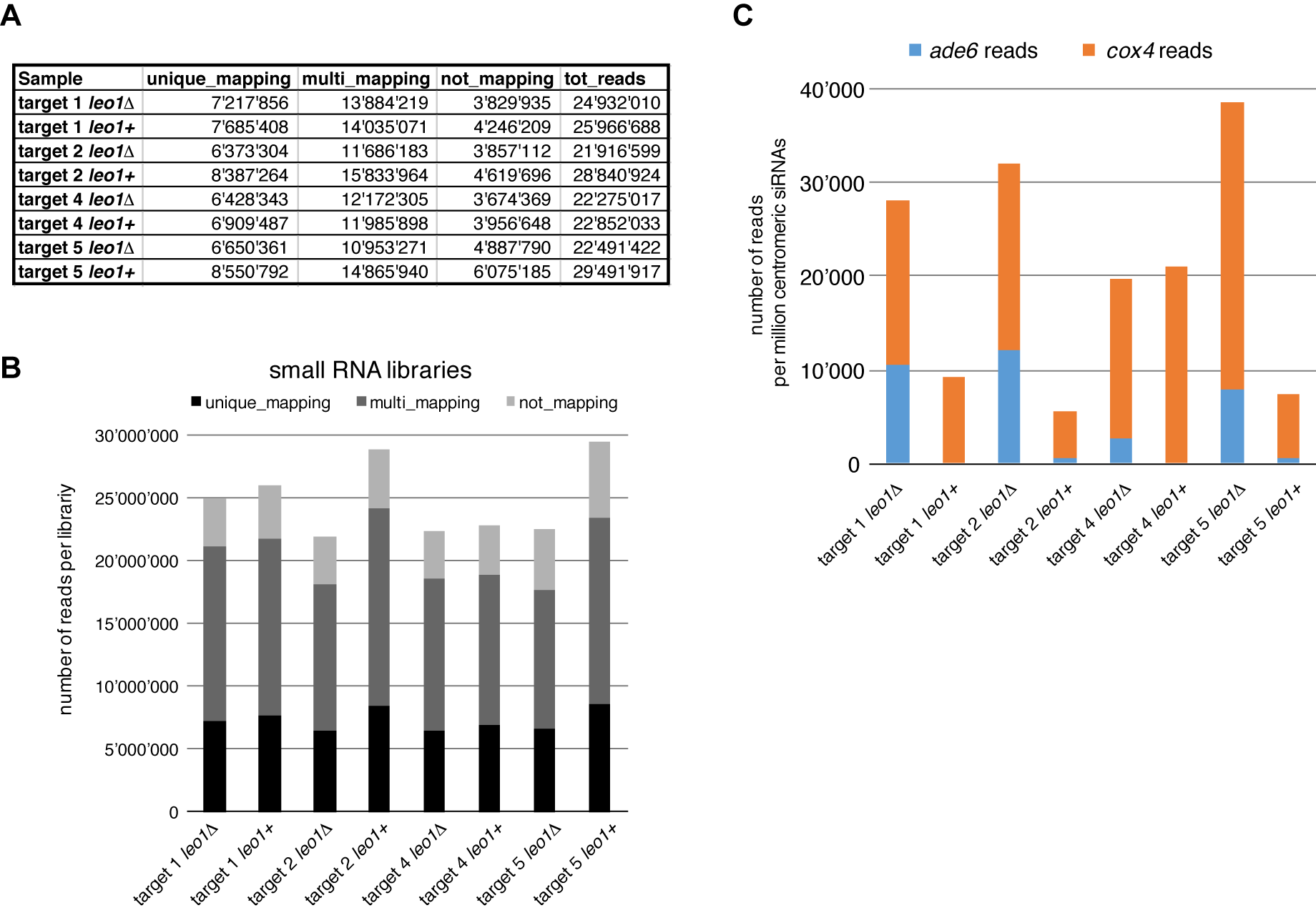


**Figure S3.** (**A**) Mapping stats of all small RNA-seq data sets used in this study. (**B**) Stacked histogram of data from A. (**C**) Stacked histogram of small RNAs mapping to *ade6+* (blue) and *cox4+* loci (orange). Note the dramatic increase of siRNAs mapping to *ade6+*, and to a lesser extent to *cox4+*, which indicates secondary siRNA generation in silencing competent *leo1Δ* cells.

**Supplemental Tables**

Table 1: Primers

| **Name** | **Sequence** | **Target** | **Comment** |
| --- | --- | --- | --- |
| mb6582 | ATAAGGTATAACGACAACAAACG | *ade6*+ promoter | primer pair 1, forward |
| mb6583 | GCATACGCTAAAATCAATATAGC | *ade6*+ promoter | primer pair 1, reverse |
| mb6584 | CGAAAAACAGGTTGTAGGGATCC | *ade6*+ CDS | primer pair 2, forward |
| mb6585 | GAATTTGCTGCATCCAAGATGATGC | *ade6*+ CDS | primer pair 2, reverse |
| mb6586 | AAACATTGGCTTACGACGGTCG | *ade6*+ CDS | primer pair 3, forward |
| mb6587 | GAACGAACTTTTCAACATAAAGCG | *ade6*+ CDS | primer pair 3, reverse |
| mb6588 | TTCCACAACTCATGCGTTGATGG | *ade6*+ CDS | primer pair 4, forward |
| mb6589 | GATGCAAAGTTGCACCGGGAATGG | *ade6*+ CDS | primer pair 4, reverse |
| mb6590 | TCACCGCACACCAGATCGCATGG | *ade6*+ CDS | primer pair 5, forward |
| mb6591 | ATACCAGGCAAATGAGCGGCACC | *ade6*+ CDS | primer pair 5, reverse |
| mb6592 | CCCTTTTGGCTGCTATGGAGAGC | *ade6*+ CDS | primer pair 6, forward |
| mb6593 | CTATGCAGAATAATTTTTCCAACC | *ade6*+ CDS | primer pair 6, reverse |
| mb6730 | gcattgaagtttaagataacattgg | *ade6*+ terminator | primer pair 7, forward |
| mb6731 | taacatagccaaacataatgcgg | *ade6*+ terminator | primer pair 7, reverse |
| mb555 | TCCTCATGCTATCATGCGTCTT | *act1*+ CDS | forward |
| mb556 | CCACGCTCCATGAGAATCTTC | *act1*+ CDS | reverse |
| mb566 | TCCGTTCCCCTCGAGGTT | *adh1*+ CDS | forward |
| mb567 | TCAAGGCACGATAGCAAGTGA | *adh1*+ CDS | reverse |
| mb2202 | CATGGAAATTGCAGTGATGGTAGT | *ade6*+ CDS | promoter distal pair, forward |
| mb167 | GGTGTTGATTTCGCTGAAAGGAAGACCAC | *ade6*+ CDS | promoter distal pair reverse |
| mb10007 | GCTGCCAAGGTATATACATAC | *ade6*+ 5’UTR | promoter proximal pair, forward |
| mb10008 | TTTTCAACACTTTCCTGGTC | *ade6*+ CDS | promoter proximal pair reverse |
| mb549 | AAGGAATGTGCCTCGTCAAATT | *cendg* | forward |
| mb550 | TGCTTCACGGTATTTTTTGAAATC | *cendg* | reverse |
| mb4848 | AGTGTGACGTTGACATCCGTAA | mouse actin CDS | forward |
| mb4849 | CTCAGGAGGAGCAATGATCTTG | mouse actin CDS | reverse |

Unless indicated in the figure legend, primer pair 3 was used for qPCR and ddPCR to amplify ade6.

**Table 2: Strains**

| **Name** | **Genotype** | **ref** |
| --- | --- | --- |
| SPB462 | *h- leu1-32 ura4-D18 nmt1+::ade6-hp+-nat* | 1 |
| SPB2383 | *h- leu1-32 ura4-D18 nmt1+::ade6-hp+-nat leo1Δ::kan* | 2 |
| SPB2562 | *h- leu1-32 ura4-D18 nmt1+::ade6-hp+-nat kan-P3nmt1::ade6+* | 2 |
| SPB2574 | *h- leu1-32 ura4-D18 nmt1+::ade6-hp+-NAT kan-P3nmt1::ade6+ leo1Δ::hph* | 2 |
| SPB2489 | *h- leu1-32 ura4-D18 ade6-target4 nmt1+::ura4-hp+-nat leo1Δ::kan* | 2 |
| SPB2491 | *h- leu1-32 ura4-D18 ade6-target4 nmt1+::ura4-hp+-nat* | 2 |
| SPB2591 | *h- leu1-32 ura4-D18 ade6-target4 leo1Δ::kan* | 2 |
| SPB2593 | *h- leu1-32 ura4-D18 ade6-target4* | 2 |
| SPB2493 | *h- leu1-32 ura4-D18 ade6-target5 nmt1+::ura4-hp+-nat leo1Δ::kan* | 2 |
| SPB2496 | *h- leu1-32 ura4-D18 ade6-target5 nmt1+::ura4-hp+-nat* | 2 |
| SPB2583 | *h- leu1-32 ura4-D18 ade6-tarfet5 leo1Δ::kan* | 2 |
| SPB2394 | *h+ leu1-32 ura4-D18 ade6-target5* | 2 |
| SPB2554 | *h- leu1-32 ura4-D18 ade6-target6 nmt1+::ura4-hp+-nat leo1Δ::kan* | 2 |
| SPB2497 | *h- leu1-32 ura4-D18 ade6-target6 nmt1+::ura4-hp+-nat* | 2 |
| SPB2584 | *h- leu1-32 ura4-D18 ade6-target6 leo1Δ::kan* | 2 |
| SPB2395 | *h+ leu1-32 ura4-D18 ade6-target6* | 2 |
| SPB2499 | *h- leu1-32 ura4-D18 ade6-target1 nmt1+::ura4-hp+-nat leo1Δ::kan* | 2 |
| SPB2502 | *h- leu1-32 ura4-D18 ade6-target1 nmt1+::ura4-hp+-nat* | 2 |
| SPB2426 | *h- leu1-32 ura4-D18 ade6-target1 leo1Δ::kan* | 2 |
| SPB2592 | *h- leu1-32 ura4-D18 ade6-taeget1* | 2 |
| SPB2503 | *h- leu1-32 ura4-D18 ade6-target2 nmt1+::ura4-hp+-nat leo1Δ::kan* | 2 |
| SPB2505 | *h+ leu1-32 ura4-D18 ade6-target2 nmt1+::ura4-hp+-nat* | 2 |
| SPB2580 | *h- leu1-32 ura4-D18 ade6-target2 leo1Δ::kan* | 2 |
| SPB2388 | *h+ leu1-32 ura4-D18 ade6-target2* | 2 |
| SPB2506 | *h- leu1-32 ura4-D18 ade6-target3 nmt1+::ura4-hp+-nat leo1Δ::kan* | 2 |
| SPB2508 | *h- leu1-32 ura4-D18 ade6-target3 nmt1+::ura4-hp+-nat* | 2 |
| SPB2581 | *h- leu1-32 ura4-D18 ade6-target3 leo1Δ::kan* | 2 |
| SPB2389 | *h+ leu1-32 ura4-D18 ade6-target3* | 2 |
| SPB2843 | *h- leu1-32 ura4-D18 ade6Δ*  III:968615:: *Pade6-ade6-Ttef1* | 2 |
| SPB2854 | *h- leu1-32 ura4-D18 ade6Δ* III:968615*:: Pade6-ade6-Ttef1 nmt1+::ade6-hp+-nat* | 2 |
| SPB2919 | *h- leu1-32 ura4-D18 ade6Δ* III:968615*:: Pade6-ade6-Ttef1 leo1Δ::kan* | 2 |
| SPB2856 | *h- leu1-32 ura4-D18 ade6Δ* III:968615*:: Pade6-ade6-Ttef1 nmt1+::ade6-hp+-nat leo1Δ::kan* | 2 |
| SPB2844 | *h- leu1-32 ura4-D18 ade6Δ *III:968615*::ade6-Ttef1* | 2 |
| SPB2864 | *h- leu1-32 ura4-D18 ade6Δ* III:968615*::ade6-Ttef1 nmt1+::ade6-hp+-nat* | 2 |
| SPB2920 | *h- leu1-32 ura4-D18 ade6Δ* III:968615*::ade6-Ttef1 leo1Δ::kan* | 2 |
| SPB2867 | *h- leu1-32 ura4-D18 ade6Δ* III:968615*::ade6-Ttef1 nmt1+::ade6-hp+-nat leo1Δ::kan* | 2 |
| SPB2845 | *h- leu1-32 ura4-D18 ade6Δ* III:968615*:: Pade6-ade6* | 2 |
| SPB2858 | *h- leu1-32 ura4-D18 ade6Δ* III:968615*:: Pade6-ade6 nmt1+::ade6-hp+-nat* | 2 |
| SPB2921 | *h- leu1-32 ura4-D18 ade6Δ* III:968615*:: Pade6-ade6 leo1Δ::kan* | 2 |
| SPB2862 | *h- leu1-32 ura4-D18 ade6Δ* III:968615*:: Pade6-ade6 nmt1+::ade6-hp+-nat leo1Δ::kan* | 2 |
| SPB2896 | *h- leu1-32 ura4-D18 ade6Δ* III:968615*:: ade6 ORF* | 2 |
| SPB2912 | *h- leu1-32 ura4-D18 ade6Δ* III:968615*:: ade6 ORF nmt1+::ade6-hp+-nat* | 2 |
| SPB2915 | *h- leu1-32 ura4-D18 ade6Δ *III:968615*:: ade6 ORF leo1Δ::kan* | 2 |
| SPB2913 | *h- leu1-32 ura4-D18 ade6Δ* III:968615*:: ade6 ORF nmt1+::ade6-hp+-nat leo1Δ::kan* | 2 |

1, Kowalik, K. M., Shimada, Y., Flury, V., Stadler, M. B., Batki, J., & Bühler, M. (2015). The Paf1 complex represses small-RNA-mediated epigenetic gene silencing*. Natu*re*, 5*20(7546), 248–252. http://doi.org/10.1038/nature14337

2, this study
